# Supplementary material for: Retrieval of Hyperspectral Information from Multispectral Data for Perennial Ryegrass Biomass Estimation
Source: Sensors (Basel). 2020 Dec 15;20(24):7192. doi: 10.3390/s20247192 (PMC7765461; doi:10.3390/s20247192)
Supplement: Supplementary file 1 [file sensors-20-07192-s001.pdf]

sensors

Retrieval of Hyperspectral Information from Multispectral Data for Perennial Ryegrass Biomass Estimation

Annex 1. Vegetation Indices Formulas

Current Method:

Normalized Difference Vegetation Index (NDVI)

$$\text{NDVI} = \frac{B_{NIR} - B_{Red}}{B_{NIR} + B_{Red}} \quad (1)$$

Green Normalized Difference Vegetation Index (GNDVI)

$$\text{GNDVI} = \frac{B_{NIR} - B_{Green}}{B_{NIR} + B_{Green}} \quad (2)$$

Normalized Difference Red Edge Index (NDRE)

$$\text{NDRE} = \frac{B_{NIR} - B_{Red\ Edge}}{B_{NIR} + B_{Red\ Edge}} \quad (3)$$

Normalized Green Red Difference Index (NGRDI)

$$\text{NGRDI} = \frac{B_{Green} - B_{Red}}{B_{Green} + B_{Red}} \quad (4)$$

Leaf Chlorophyll Index (LCI)

$$\text{LCI} = \frac{B_{NIR} - B_{Red\ Edge}}{B_{NIR} + B_{Red}} \quad (5)$$

Structure Intensive Pigment Index 2 (SIPI2)

$$\text{SIPI2} = \frac{B_{NIR} - B_{Green}}{B_{NIR} - B_{Red}} \quad (6)$$

Proposed Method:

Optimized Normalized Ration index- refer to O. Mutanga & Skidmore, (2004):

$$\text{Opt NRI} = \frac{B_{755} - B_{745}}{B_{755} + B_{745}} \quad (7)$$

Normalized Band Depth Index -refer to Mutanga & Skidmore (2004):

$$\text{NDBI}_{\lambda} = \frac{BD_{\lambda} - Dc}{BD_{\lambda} + Dc} \quad (8)$$

Band Area:

$$\text{Band Area} = \int_{550}^{790} 1 - BD \quad (9)$$

For the calculation of these indices refer to https://cran.r-project.org/web/packages/hsdar/ .

References:

1. Mutanga, O.; Skidmore, A. K. Narrow band vegetation indices overcome the saturation problem in biomass estimation. *International Journal of Remote Sensing*, **2004,** *25(19)*, 3999–4014. https://doi.org/10.1080/01431160310001654923
2. Mutanga, O.; Skidmore, A. K. Hyperspectral band depth analysis for a better estimation of grass biomass (Cenchrus ciliaris) measured under controlled laboratory conditions. *International Journal of Applied Earth Observation and Geoinformation*, **2004,** *5(2)*, 87–96. https://doi.org/10.1016/j.jag.2004.01.001

Publisher's Note: MDPI stays neutral with regard to jurisdictional claims in published maps and institutional affiliations.

© 2020 by the authors. Licensee MDPI, Basel, Switzerland. This article is an open access article distributed under the terms and conditions of the Creative Commons Attribution (CC BY) license (http://creativecommons.org/licenses/by/4.0/).
